# Mapping Multi-Modal Fatigue in Elite Soccer Through Sweat-Omics Perspectives: A Narrative Review

**DOI:** 10.3390/biology14081069

**Published:** 2025-08-16

**Authors:** Moses Gnanasigamani, Ersan Arslan, Yusuf Soylu, Bulent Kilit, Paweł Chmura

**Affiliations:** 1 Department of Individual and Team Sports, Wroclaw University of Health and Sport Sciences, 51-612 Wrocław, Poland; moseshubertsingh05@gmail.com; 2 Faculty of Sport Sciences, Tokat Gaziosmanpasa University, Tokat 60250, Turkey; ersan.arslan@gop.edu.tr (E.A.); yusuf.soylu@gop.edu.tr (Y.S.); bulent.kilit@gop.edu.tr (B.K.)

**Keywords:** sweat biomarkers, fatigue monitoring, soccer physiology, wearable sensors, omics profiling

## Abstract

Fatigue in elite soccer is a multifaceted phenomenon involving physical, metabolic, psychological, and neuromuscular stressors that accumulate over training and competition. Traditional monitoring tools, while informative, are often invasive, impractical during play, or fail to provide real-time insights. This narrative review synthesizes sweat-based biomarkers linked to fatigue in elite soccer, with a focus on multi-modal domains (neuromuscular, metabolic, inflammatory, psychological). Using an integrative approach, we reviewed studies published across databases including PubMed, Scopus, and Web of Science that evaluate sweat biomarkers of different types of fatigue in soccer. We identified key candidates—lactate, sodium, cortisol, IL-6, and hypoxanthine—and evaluated their potential in non-invasive monitoring. Youth-specific and female populations remain underrepresented. We also assessed each biomarker’s technological maturity using a sweat-specific Technology Readiness Level (TRL) framework. Based on these findings, we propose a translational framework for practitioners and outline priorities for future research and validation in real-world sport settings.

## Simple Summary

Fatigue in elite soccer players is complex and influenced by both physical and mental factors. When not properly monitored, it can lead to reduced performance, greater risk of injury, and long-term health problems, especially in young athletes. This review explores how sweat—the fluid naturally released by the body—can be used to monitor different types of fatigue in real time during training and games. By using advanced wearable sensors and tools that analyze the small molecules and signals in sweat, researchers and coaches may soon be able to track changes in hydration, energy use, stress, and muscle function without needing blood samples or lab tests. The goal of this study is to summarize what is currently known about sweat-based monitoring, highlight the most promising sweat markers of fatigue, and identify what gaps still exist in science. By using a non-invasive and practical approach, sweat-based monitoring may offer a new way to keep athletes healthier, more prepared, and performing at their best.

## 1. Introduction

Fatigue in soccer is a multifaceted and complex phenomenon resulting from the dynamic interaction of neuromuscular, metabolic, psychological, and physiological systems. These interacting domains collectively contribute to performance decrements and elevate the risk of injury—particularly among youth athletes undergoing growth spurts, hormonal fluctuations, and progressively increasing training demands [1,2]. In contrast to adult professionals, elite youth soccer players possess distinct developmental vulnerabilities, including musculoskeletal immaturity, heightened sensitivity to training stimuli, and an elevated risk of non-contact injuries, stress fractures, and overtraining [1]. Moreover, these athletes are subject to mounting competitive demands and congested match schedules; however, prevailing fatigue-monitoring protocols are seldom tailored to account for their unique physiological and maturational profiles.

Conceptually distinct from transient physical exhaustion, fatigue in elite soccer reflects a systems-level dysfunction across multiple physiological axes [3,4]. Neuromuscular fatigue entails reductions in force-generating capacity arising from both central motor drive impairment and peripheral excitation–contraction failure [5]. Metabolic fatigue emerges from perturbations in substrate availability and intramuscular accumulation of by-products such as lactate and ammonia that disrupt pH balance and enzymatic kinetics [6]. Psychological fatigue encompasses cognitive overload, stress-induced motivational decline, and affective dysregulation that compromise tactical decision-making and attentional control [7]. Together, these modalities culminate in measurable decrements in sprint capacity, technical execution, and tactical cohesion during match-play [8].

Conventional methods for assessing fatigue—such as GPS-derived external load metrics, heart rate monitoring, and subjective ratings of perceived exertion—provide useful estimates of overall workload but are inherently indirect and temporally dissociated from real-time internal physiological disruptions [9,10]. Although venipuncture-based assays measuring biomarkers such as lactate, creatine kinase (CK), and cortisol yield more direct biochemical indicators of fatigue, their invasive nature, financial cost, and operational complexity limit their feasibility for frequent, field-based application, particularly in youth athletic populations [11]. In light of the demand for ecologically valid, non-invasive, and developmentally appropriate assessment tools, sweat has emerged as a promising alternative biofluid for real-time, in-field monitoring of fatigue in elite youth soccer players.

Beyond its canonical thermoregulatory function, sweat contains a diverse repertoire of molecular constituents—including electrolytes, metabolites, peptides, and trace elements—that reflect acute physiological stress and chronic training adaptation [12]. The innovation lies not merely in analyte detection, but in the advent of high-resolution, integrative approaches that capture complex biomolecular signatures in sweat. To conceptualize this paradigm, the term “sweat-omics” is herein defined as the integrative, high-resolution profiling of sweat-derived molecular constituents—spanning metabolomics, proteomics, lipidomics, mineralomics, and emerging omics layers—using advanced analytical and wearable sensor platforms to dynamically map physiological states associated with fatigue, stress, and recovery in sport-specific contexts.

Unlike traditional single-analyte sweat monitoring, sweat-omics adopts a systems-biology lens, capturing the multidimensional signatures of fatigue across neuromuscular, endocrine, immune, and metabolic domains [13,14]. Advances in wearable microfluidic platforms now enable on-body, continuous acquisition of sweat analytes during match and training contexts, bridging the translational gap between molecular diagnostics and applied sport practice [11,15]. This represents a significant step forward in fatigue monitoring—particularly in youth athletes—where invasive sampling is ethically challenging, and subjective measures are prone to bias.

In soccer players, sweat-based detection of lactate and ammonia has been shown to approximate anaerobic glycolytic flux, while sodium and potassium concentrations reflect ion loss critical for membrane excitability and muscular contractility [16]. Cortisol levels in sweat track hypothalamic–pituitary–adrenal (HPA) axis activation, correlating with psychophysiological stress during competition. Yet, despite these advances, the application of sweat-omics remains conspicuously underdeveloped in elite youth soccer—where biological maturation, load sensitivity, and injury vulnerability necessitate highly individualized fatigue profiling strategies.

This narrative review seeks to map the interdependent domains of fatigue in elite youth soccer through the lens of sweat-omics, synthesizing current evidence while identifying both research gaps and practical limitations to inform future innovation. Effective implementation of sweat-based monitoring in soccer first requires a clear understanding of the underlying fatigue modalities experienced by athletes. By delineating these interconnected domains—neuromuscular, metabolic, psychological, and physical—it becomes possible to identify the physiological processes that relevant sweat biomarkers should capture. Establishing a robust conceptual framework for fatigue in elite soccer is therefore essential to guide the mapping of specific sweat-derived analytes to their corresponding fatigue mechanisms.

As a narrative review, this manuscript does not adhere to a rigid systematic review protocol. Instead, it employs an iterative, multistrand research weaving methodology that integrates structured database searches, conceptual clustering, and thematic synthesis. The initial literature identification phase drew upon multiple databases—including Google Scholar, Scopus, Web of Science, and PubMed—and was refined through successive rounds of iterative filtering. Three complementary search streams were employed: (1) “sweat” AND (“soccer” OR “football” OR “futsal”) to identify sport-specific applications; (2) “sweat metabolomics” AND (“athletes” OR “exercise” OR “sports”) to capture broader technological and physiological insights; and (3) “sweat” AND “fatigue” AND (“metabolic” OR “physical” OR “neuromuscular” OR “psycho*”) to identify mechanistic and clinical relevance. These were supplemented by targeted searches for individual analytes (hypoxanthine, pyruvate).

This non-linear, exploratory methodology reflects the nature of an emerging interdisciplinary field. The science of sweat biomarkers in high-performance sport lacks a consolidated evidence base, and soccer-specific applications remain limited. Accordingly, this manuscript offers not a systematic enumeration of studies, but rather a targeted synthesis of mechanistic evidence, practical use cases, and biomarker feasibility as they pertain to the multifaceted fatigue demands of elite soccer.

## 2. Fatigue in Elite Youth Soccer: A Conceptual Framework

**Figure 1:**
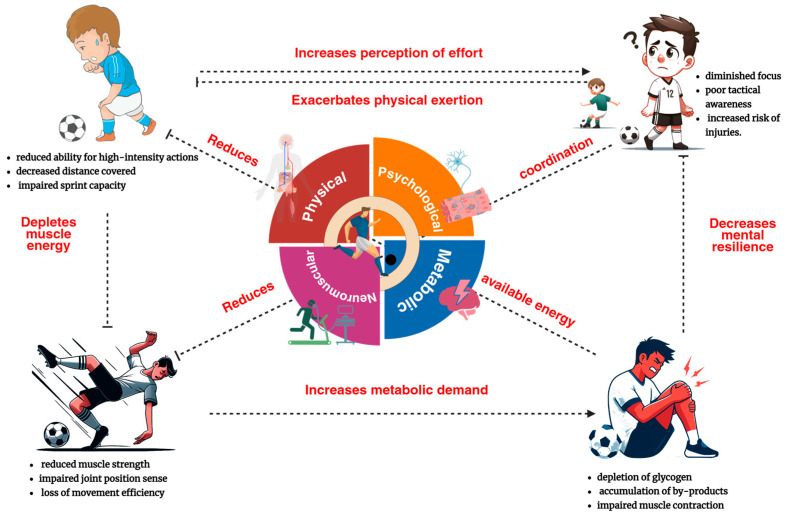
Interconnected fatigue domains in elite youth soccer and their physiological impacts. This conceptual diagram illustrates the four primary fatigue domains—neuromuscular, metabolic, psychological, and physical—and how they interact to affect performance and injury risk in elite youth soccer.

Elite soccer imposes a multidimensional physiological and cognitive workload characterized by high-frequency accelerations, decelerations, rapid directional changes, complex perceptual–motor demands, and sustained psychomotor vigilance. These cumulative stressors elicit a heterogeneous fatigue response across neuromuscular, metabolic, thermoregulatory, and neurocognitive systems, emerging from both central (cortical/spinal) and peripheral (muscular and metabolic) origins (Figure 1) [17]. Central fatigue is operationally defined as a decrement in voluntary motor output stemming from altered central nervous system excitability, neurotransmitter dysregulation (serotonin–dopamine imbalance), and reduced motoneuron drive [18]. Peripheral fatigue, by contrast, is driven by impairments at or distal to the neuromuscular junction—including excitation–contraction coupling dysfunction, intracellular ionic disturbances (K^+^, Na^+^, Ca^2+^), sarcoplasmic reticulum fatigue, and accumulation of metabolic by-products that disrupt actomyosin cross-bridge cycling [3].

In parallel, cognitive or psychogenic fatigue arises from sustained attentional demands, decision-making under temporal constraints, and exposure to environmental stressors such as competitive anxiety and crowd noise. These factors collectively impair executive function and reduce attentional bandwidth. Additionally, thermal and hydration-related fatigue, triggered by elevated core body temperature and sweat-induced fluid and electrolyte losses, disrupts cardiovascular homeostasis and lowers neuromuscular excitability thresholds. Even mild dehydration (exceeding 2% of body mass) has been shown to impair both physical performance and cognitive task execution, particularly in thermally stressful conditions [19,20]. Over time, insufficient recovery from cumulative training and match-induced physiological strain can lead to non-functional overreaching or the development of overtraining syndrome—multisystemic conditions marked by immune dysregulation, neuroendocrine maladaptation, and persistent performance decrements [4].

Within this complex, multifactorial landscape, neuromuscular fatigue constitutes a primary domain, encompassing impairments in both central motor drive and peripheral muscle contractility. In elite youth soccer, frequent high-intensity actions such as sprinting, tackling, and jumping impose sustained neuromuscular stress, resulting in observable reductions in sprint performance and vertical jump height in second-half periods [5]. These effects are further amplified in youth athletes due to ongoing developmental processes, including the maturation of neuromuscular coordination, motor unit recruitment efficiency, and endocrine function [21], highlighting the need for age-appropriate and developmentally sensitive monitoring strategies.

Metabolic fatigue is defined by depletion of intramuscular energy substrates—most notably phosphocreatine and glycogen—and the accumulation of fatigue-inducing metabolites including lactate, hydrogen ions, and ammonia [22]. Repeated high-intensity efforts in soccer depend on rapid ATP resynthesis via glycolysis and oxidative phosphorylation. As match duration increases, particularly under congested fixtures, glycogen depletion—especially in type II fibers—degrades repeated sprint ability and explosive power output [23]. Youth players, with inherently lower glycogen stores and immature enzymatic capacities (phosphofructokinase, citrate synthase), exhibit heightened susceptibility to metabolic fatigue [24]. Non-invasive sweat biomarkers such as lactate and ammonia have shown promise in reflecting these anaerobic metabolic perturbations, providing potential surrogates for internal metabolic load in real time [12,13].

Psychological fatigue encompasses the decline in neurocognitive and emotional resources essential for decision-making, attentional control, and motivation [25]. Match-induced psychogenic fatigue manifests as slowed reaction time, suboptimal tactical choices, and elevated error rates under pressure [7]. These effects are mediated in part by stress-induced hormonal shifts—particularly elevated cortisol via HPA axis activation—which alter central arousal and mood state. In adolescent athletes, psychosocial stressors (parental expectations, academic burdens, identity formation) further amplify vulnerability to psychological fatigue [26]. Emerging biosensing technologies incorporating cortisol and cytokine (IL-6) detection in sweat enable real-time psychophysiological fatigue profiling, bridging objective hormonal data with subjective cognitive load [11,15].

Physical fatigue, the integrated manifestation of the above domains, is typically quantified through reductions in external load (total distance, high-speed running) and sport-specific technical metrics (pass accuracy, duels won). Time-motion analyses in youth soccer consistently demonstrate second-half decrements in high-intensity locomotor output [27], paralleled by increases in technical execution errors and reduced tactical coherence [28]. Importantly, physical fatigue should not be regarded as an isolated phenomenon, but rather as the phenotypic expression of cumulative internal load, shaped by a complex interplay of environmental conditions and individual physiological variability.

Beyond these core domains, thermal and hydration-related fatigue emerges from uncompensated heat stress and sweat-derived fluid and electrolyte losses. Progressive hypohydration and sodium-chloride depletion reduce plasma volume, elevate cardiovascular strain, and impair central thermoregulatory control. Variability in sweat electrolyte concentrations—modulated by acclimatization status, glandular site, and genetic predisposition—complicates direct interpretation but remains a viable parameter for individualized hydration strategies [13,19].

Chronic fatigue and overtraining represent cumulative maladaptive states driven by repetitive training stimuli in the absence of sufficient recovery. These syndromes are characterized by sustained declines in performance, mood disturbances, and biochemical markers of immune and endocrine dysregulation. Although acute fatigue monitoring is gaining momentum in sports science, there remains a notable absence of sweat-based methodologies for longitudinal tracking of cumulative training load or for the early detection of overtraining syndrome (OTS), a critical concern in elite youth soccer, where delayed recovery and developmental stressors frequently intersect. Although overtraining-related immune and endocrine disturbances have been studied in blood, the sweat-based detection of such chronic biomarkers remains largely unexplored in soccer contexts. There is currently no evidence supporting the use of sweat biomarkers—such as prolonged cortisol elevation or persistent cytokine expression—as indicators of OTS onset in elite soccer players, representing a major translational gap. Sweat-based detection of stress and inflammatory biomarkers such as cortisol and IL-6 offers non-invasive insights into systemic strain and recovery status, albeit with current limitations in standardization and validation [4,11,15].

In the context of elite youth soccer, where somatic growth, neuroendocrine maturation, and training load interact dynamically, a robust conceptual framework for fatigue monitoring must be multidimensional and developmentally sensitive. Recognizing the interaction between fatigue domains is not merely academic—it is operationally critical for designing load management strategies that prevent injury, optimize adaptation, and support long-term athlete development. While blood-based biomarkers and external workload metrics offer partial visibility into fatigue dynamics, they lack the integration, granularity, and practicality required for field-based monitoring in youth populations This underscores a compelling rationale for the investigation of sweat as a high-resolution, non-invasive biofluid capable of capturing multidomain fatigue signatures in real time. The subsequent section critically examines the physiological validity, technological feasibility, and sport-specific applicability of sweat biomarkers in the context of fatigue monitoring.

The following section critically evaluates sweat as a biofluid with the capacity to non-invasively capture real-time physiological signals across multiple fatigue domains. Each fatigue domain may be monitored by different analytes in sweat, captured via omics platforms or wearable sensors (Table 1).

## 3. Sweat as a Biomarker Medium

In response to the growing demand for practical, non-invasive, and athlete-friendly monitoring tools, sweat has emerged as a promising alternative to conventional biofluid sampling methods. Unlike blood collection, sweat sampling is better suited for real-time application during training and competition, particularly within youth and team sports [4,11,13]. We explore the utility of sweat as a biomarker-rich medium for monitoring multiple dimensions of physiological fatigue.

Sweat is an easily accessible, externally secreted fluid produced by eccrine glands, containing a complex matrix of electrolytes, metabolites, proteins, hormones, and trace elements reflective of systemic physiological states [37]. Electrolytes such as sodium (Na^+^), chloride (Cl^−^), and potassium (K^+^) dominate sweat composition and are sensitive indicators of hydration status and thermal fatigue. Dehydration exceeding 2% of body mass has been shown to impair cognitive and physical performance [19]. Accordingly, sweat-based measurements of electrolyte loss therefore enable individualized fluid-replacement strategies critical for fatigue mitigation, especially in hot or high-intensity environments [13].

In addition to electrolytes, sweat contains a diverse array of metabolites such as lactate, ammonia, urea, and amino acids, which reflect muscular and metabolic stress. Sweat lactate concentrations have been shown to closely mirror blood lactate dynamics during exercise, increasing in response to high-intensity anaerobic activity and providing a practical, non-invasive indicator of metabolic fatigue [12]. Similarly, sweat ammonia and urea levels indicate protein catabolism and anaerobic stress, making them valuable for profiling fatigue in sports requiring repeated sprints or prolonged high-intensity play [13]. Recent metabolomics studies have used advanced mass spectrometry techniques to detect hundreds of sweat metabolites, identifying pathways associated with energy metabolism and fatigue, such as purine degradation (hypoxanthine) and amino acid turnover [12,38].

Sweat also provides a window into psychological and neuroendocrine fatigue through hormone and cytokine profiling. Cortisol is a well-established stress biomarker measurable in sweat, with levels correlating to systemic stress responses and mental fatigue [11]. Wearable electrochemical sensors capable of real-time sweat cortisol monitoring have been developed, offering potential for in-field stress and recovery monitoring in athletes [11,15]. Similarly, sweat cytokines such as interleukin-6 (IL-6) may reflect inflammation associated with overreaching or overtraining syndromes, providing a non-invasive alternative to blood sampling for monitoring chronic fatigue [3].

Importantly, recent technological advances in wearable biosensing have transformed sweat analysis from lab-based sampling to continuous, on-body monitoring. Flexible, skin-conformal patches equipped with microfluidics and electrochemical sensors can measure multiple analytes simultaneously, including lactate, glucose, sodium (Na^+^), potassium (K^+^), and cortisol, during exercise [16]. These platforms enable athletes and coaches to receive real-time feedback on hydration status, metabolic stress, and psychological load during training and competition [11]. Gao et al. (2016) demonstrated a wearable patch that simultaneously tracks sweat lactate, glucose, and electrolytes, underscoring the potential for integrated real-time fatigue monitoring in sports [12].

Despite its considerable promise, sweat biomarker monitoring faces both methodological and physiological challenges. Sweat composition varies by glandular location, secretion rate, and environmental conditions (temperature, humidity), necessitating rigorous standardization protocols to ensure data reliability [13]. Additionally, analyte concentrations in sweat often differ from corresponding blood values, requiring careful interpretation and the establishment of sweat-specific physiological thresholds.

However, recent advances in analytical sensitivity and the development of multiplexed biosensor platforms capable of detecting multiple biomarkers simultaneously are advancing sweat-based monitoring as a powerful tool for individualized fatigue assessment. The following section explores key classes of sweat biomarkers and their relevance to distinct fatigue domains—including metabolic, neuromuscular, psychological, thermal, and inflammatory dimensions.

### 3.1. Metabolites in Sweat and Fatigue

Metabolic fatigue results from energy substrate depletion and the accumulation of metabolic by-products during intense exercise. Sweat metabolite profiling offers a non-invasive means of capturing these biochemical perturbations, with key analytes including lactate, ammonia, and hypoxanthine. In soccer players, whose bioenergetic systems are repeatedly taxed due to high-intensity intermittent activity, the relevance of these biomarkers becomes highly context-dependent and sport-specific.

Lactate, a hallmark of anaerobic metabolism, is a well-established indicator of metabolic fatigue. It accumulates in muscle and blood during strenuous effort, with a fraction appearing in sweat—via both passive diffusion and local production within the sweat glands [39,40]. Okawara et al. (2022) observed altered sweat lactate kinetics in fatigued individuals, demonstrating that post-exercise sweat lactate peaked and plateaued significantly earlier compared to rested states [41]. This accelerated onset suggests fatigue-induced modifications in lactate transport or glandular secretion [42]. Consequently, sweat lactate may serve as a proxy for lactate threshold and anaerobic stress. However, its interpretation is complex, as sweat lactate concentrations do not always correlate linearly with blood levels. At elevated sweat rates, lactate concentration may paradoxically decrease despite rising blood lactate, likely due to dilution and limited transepithelial transport. Thus, while elevated sweat lactate may denote high glycolytic flux, its absence at peak exertion must be contextualized by sweat rate and glandular kinetics.

Despite its promise, sweat lactate remains unvalidated in soccer-specific contexts. Current empirical evidence is predominantly derived from endurance sports such as running and cycling in runners and cyclists. No current evidence exists for sweat lactate validation in elite soccer cohorts. Given soccer’s reliance on repeated sprint efforts interspersed with brief recovery, future investigations should prioritize real-time lactate tracking in sweat during match-play and structured training.

Ammonia is another metabolite with well-established mechanistic links to exercise-induced fatigue. It is generated via amino acid deamination and the deamination of AMP during ATP catabolism—both of which are upregulated during prolonged or high-intensity exercise [43]. Elevated blood ammonia, historically associated with fatigue as early as the 1920s, has been implicated in central fatigue pathways, given its capacity to cross the blood–brain barrier and perturb neural signaling. In peripheral tissues, ammonia accumulation reflects increased protein catabolism and nitrogen load—biomarkers of metabolic stress or glycogen depletion. Sweat glands serve as an auxiliary route for ammonia excretion, and sweat ammonia concentrations often exceed those in plasma, implying both systemic clearance and local production [11,42]. Anecdotal athletes reports of a pungent ammonia odor in “late exercise” sweat, likely reflects the endogenous proteolysis. Interestingly, children and adolescents excrete disproportionately higher ammonia concentrations in sweat, indicating possible developmental differences in nitrogen metabolism and excretion [44].

Despite its physiological salience, ammonia has not yet been systematically quantified in sweat from soccer players. Available studies have primarily assessed ammonia or related nitrogenous compounds (urea, urate) in urine or plasma. Prado et al. (2017) applied a sport-omics framework to semi-professional soccer players and identified post-match elevations in urinary urea and urate, signaling protein catabolism and purine turnover [45]. However, ammonia was not assessed in sweat, highlighting a critical translational gap. Although theoretically plausible as a non-invasive biomarker of metabolic fatigue, ammonia remains unvalidated in soccer-specific sweat applications. Targeted validation studies that account for exercise modality, sweat rate, developmental stage, and glandular variation are urgently warranted.

Advances in metabolomics have further expanded the catalog of sweat-based fatigue biomarkers. Meihua et al. (2023) identified purine metabolites, particularly hypoxanthine, as highly sensitive to fatigue in runners [30]. Post high-intensity interval training, hypoxanthine levels in sweat increased substantially, reflecting ATP degradation. Similarly, pyruvate—a glycolytic intermediate—showed dynamic pre- versus post-fatigue alterations, providing insight into shifts in metabolic pathway dominance (aerobic vs. anaerobic) with exertion.

In soccer, hypoxanthine elevation has been consistently observed, though primarily in urine or plasma. A non-targeted metabolomic study of semi-professional players reported significantly increased urinary hypoxanthine following match-play, consistent with purine catabolism and AMP deamination [45]. Alzharani et al. (2020) further demonstrated hypoxanthine elevation in plasma of Saudi youth soccer players post training, alongside perturbations in acylcarnitines and fatty acid oxidation pathways [46]. However, to date, no evidence exists for hypoxanthine detection in sweat within soccer-specific contexts. While findings from plasma and saliva analyses support its physiological relevance, its translation to sweat-based applications remain unexplored. This represents a critical biomarker gap; hypoxanthine is validated in soccer via blood and urine but not yet through sweat-based methodologies. Bridging this gap could facilitate real-time, non-invasive fatigue monitoring.

Similarly, although pyruvate has been investigated in sweat within general populations, its application in soccer remains understudied. Studies using plasma and saliva from soccer athletes have reported training- or match-induced shifts in glycolytic intermediates, including pyruvate [17,18]. As a central node between glycolysis and mitochondrial metabolism, pyruvate may serve as a valuable fatigue biomarker. Its detection in sweat would provide insight into metabolic flexibility and substrate utilization. However, no current evidence exists for pyruvate quantification in sweat among elite soccer populations. Given the sport’s intermittent high-intensity demands, tracking pyruvate in sweat may offer real-time insight into metabolic stress if integrated into sensor-based platforms.

This underrepresentation is further substantiated in broader systematic evaluations. Pedroso et al. (2025) reviewed 21 metabolomics studies on soccer athletes; the overwhelming majority used urine, plasma, or saliva, with sweat notably underutilized [47]. These findings collectively support the conceptualization of a multimarker fatigue panel in sweat-comprising glycolytic end-products (lactate, pyruvate), nitrogenous wastes (ammonia, urea), and purine derivatives (hypoxanthine, uric acid)—each mapping to distinct metabolic axes of fatigue.

Monitoring such markers in soccer players may enable earlier detection of overtraining, optimize load management, and support individualized recovery protocols. Yet, significant gaps persist. Several metabolites well-characterized in blood or urine remain unverified in sweat. Moreover, studies stratifying biomarker dynamics across competitive levels (youth, semi-professional, elite) are lacking. In addition, while fatigue-related cytokines such as cortisol and IL-6 have been explored via serum and saliva in soccer cohorts [18], their detection in sweat—and relevance to pitch-side diagnostics—requires targeted investigation.

Although sweat-based fatigue monitoring holds significant translational potential for application in soccer, its current empirical foundation remains insufficiently developed. Future research must prioritize on soccer-specific validation of sweat-derived biomarkers, employing gold-standard calibration methods, accounting for anatomical sweat gland variability, and correcting for sweat rate confounders. Longitudinal field studies incorporating load metrics, positional demands, and environmental factors will be pivotal in advancing *sweat-omics* into routine practice in elite soccer.

### 3.2. Electrolytes in Sweat and Thermal Fatigue

Thermal and hydration-related fatigue is intrinsically linked to fluid and electrolyte loss incurred during exercise, particularly under heat stress. Electrolytes, chiefly sodium (Na^+^) and chloride (Cl^−^), with smaller contributions from potassium (K^+^), calcium (Ca^2+^), and magnesium (Mg^2+^), constitute the primary ionic components of sweat and are closely associated with both thermoregulatory and neuromuscular fatigue pathways. In soccer, prolonged exertion under warm conditions can result in substantial sweat loss, leading to significant concurrent loss of electrolytes. If fluid and electrolyte deficits are not adequately replenished, resultant dehydration and electrolyte imbalances may induce elevated cardiovascular strain, hypotension, impaired thermoregulation, and neuromuscular dysfunction—all of which contribute to performance decrements commonly referred to as thermal or heat-related fatigue.

In soccer players, these effects are particularly relevant due to the intermittent nature of high-intensity running and often warm, humid match environments. A recent study by Suarez-Ortegón et al. (2024) of professional Colombian male soccer players reported a mean sweat rate of ~1.7 L/h and forearm sweat [Na^+^] of ~27 mmol/L (~0.6 g Na^+^/L), equating to approximately 1.0 g of sodium lost per hour of play [32]. Players with higher muscle and body mass exhibited higher sweat sodium concentrations and total Na^+^ losses, reinforcing the value of individualized sweat profiling for fatigue mitigation. By contrast, sweat sodium values in runners often exceed 50 mmol/L, suggesting sport-specific differences in sweat gland activity or adaptation. These results underscore that sodium loss in sweat is both athlete- and sport-dependent.

Whereas, in female soccer players, the threshold for performance impairment due to dehydration may be lower. A mere 2% loss in body mass from sweating can degrade aerobic capacity and ball control skills. Consequently, strategic hydration protocols and electrolyte supplementation are especially critical in the women’s game.

Quantifying sweat electrolyte loss during training or competition enables the personalization of rehydration strategies, potentially delaying the onset of heat-induced performance decline. In high-temperature environments, elite soccer players may lose between 1 and 2 L of sweat per hour, corresponding to sodium losses of approximately 1.5–3.0 g per hour. Without adequate replacement, such deficits can impair muscle excitability and neuromuscular transmission, contributing to exercise-associated muscle cramps and premature fatigue [39,40].

It is important to emphasize that sweat electrolyte concentrations are not direct indicators of whole-body hydration status or fatigue level. Sweat sodium ([Na^+^]) and chloride ([Cl^−^]) levels are modulated by both sweat rate and glandular reabsorption processes. As exercise intensity and sweating increase, sweat [Na^+^] and [Cl^−^] tend to rise (i.e., the sweat becomes saltier at higher sweat rates) [42]. In contrast, blood plasma sodium concentrations remain relatively stable or may even increase during dehydration due to hemoconcentration. Consequently, there is no linear or one-to-one correlation between sweat electrolyte concentrations and systemic electrolyte status.

Potassium exhibits a distinct physiological pattern: sweat potassium ([K^+^]) concentrations are typically lower than intracellular or plasma levels, and unlike sodium, [K^+^] in sweat tends to decline during high-intensity exercise. This occurs even as plasma [K^+^] transiently decreases, driven by muscular uptake during recovery phases.

These dissociations underscore that sweat electrolyte composition is, to a large extent, governed by local glandular regulation and is not a direct proxy for plasma electrolyte homeostasis. In other words, elevated sweat [Na^+^] does not necessarily imply systemic sodium deficiency. It may instead reflect a high sweat rate or reduced ductal reabsorption. Furthermore, sweat composition varies by anatomical site and collection method. Malefo et al. (2025) demonstrated that regional patch-based sweat collection (forearm) in elite South African soccer players yielded [Na^+^] values ranging from 12.1 to 54.5 mmol/L, with substantial intra-individual variability [31]. This variability complicates the use of sweat sodium as a definitive fatigue marker across sessions or populations.

Critically, there is currently no validated threshold of sweat sodium concentration that correlates directly with in-game fatigue or performance decrements in elite soccer populations. While such associations have been more clearly established in endurance sports, they remain unsubstantiated in soccer-specific contexts. Thus, sweat [Na^+^] should not yet be interpreted as a direct index of fatigue in the sport. Nevertheless, total electrolyte loss via sweat remains mechanistically linked to fatigue potential. Excessive sodium chloride (NaCl) loss, without adequate replacement, can result in extracellular fluid shifts, impaired neuromuscular transmission (muscle cramps), and, in severe cases, exercise-associated hyponatremia—all of which can exacerbate fatigue-related symptoms.

In elite soccer settings, practitioners often monitor players’ pre- and post-training or match-play body mass changes and, in some cases, assess sweat sodium concentrations during training sessions to estimate individual salt loss. This data is used to ensure players begin matches well-hydrated and receive electrolyte-rich fluids at half-time or during cooling breaks. By doing so, they proactively delay the onset of hyperthermia and preserve neuromuscular function and cognitive performance in the final phases of competition.

While sweat electrolyte loss is mechanistically linked to thermal fatigue and has clear relevance in soccer-specific exertional settings, it is best viewed as a preventative biomarker guiding hydration interventions rather than a validated real-time index of fatigue. The need for standardized collection protocols and validation studies in elite soccer populations remains a critical gap in the literature.

### 3.3. Hormonal Markers in Sweat and Stress Fatigue

The neuroendocrine dimension of fatigue, particularly psychological and central fatigue, is increasingly accessible through sweat biomarkers, most notably cortisol, a canonical marker of hypothalamic–pituitary–adrenal (HPA) axis activation. Cortisol synthesis, triggered by adrenocorticotropic hormone (ACTH) release, responds to both physiological exertion and psychological load, making it uniquely positioned to index the bidirectional stress encountered in elite sport. In this context, sweat analysis offers a minimally invasive modality for continuous cortisol profiling, as sweat concentrations of cortisol are broadly proportional to free serum levels [39,40].

Cortisol detection in sweat has been reliably demonstrated in controlled laboratory settings. For instance, during continuous cycling protocols, sweat cortisol levels rise progressively, peaking approximately 40 min into sustained exertion, with a subsequent plateau or attenuation reflecting negative feedback inhibition of the HPA axis. This temporal pattern aligns with canonical endocrine response curves and underscores the viability of sweat sampling for stress monitoring in aerobic modalities. Pearlmutter et al. (2020) compared sweat and saliva cortisol kinetics in exercising individuals and observed that apocrine sweat contained higher concentrations than saliva, particularly in males, further supporting the translational value of sweat-based metrics for field sports [48].

In soccer players, Nunes et al. (2021) provided critical empirical support for the use of sweat cortisol as a valid fatigue marker [49]. In their analysis of eccrine sweat samples following a 90 min training game, both cortisol (hydrocortisone) and cortisone were elevated post session, reflecting robust HPA-axis engagement in response to intermittent high-intensity load. Further corroborating these findings, Malefo et al. (2025), in a sweat metabolomics study of professional soccer players in Pretoria, South Africa, identified cortisol-related metabolites as components of the post-training metabolic fingerprint, further establishing the contextual relevance of sweat cortisol in soccer physiology [31].

However, despite the growing body of supportive evidence, no validated sweat cortisol thresholds currently exist for predicting performance decline, overreaching, or recovery status in elite soccer populations. Moreover, considerable inter-individual variability in sweat gland density, anatomical sampling site, and environmental stressors (humidity, skin temperature) complicate cross-sectional interpretation. Sweat cortisol kinetics may also diverge from plasma or saliva due to time lag, local metabolism, and glandular reabsorption, posing significant challenges for calibration against canonical “gold-standard” matrices.

Sweat cortisol should thus be contextualized not merely as a proxy for systemic stress but as a composite index reflecting both glandular function and the integrated psychological–physiological load experienced by the athlete [50]. In soccer contexts, where athletes face repeated cognitive stressors (tactical decisions, crowd pressure) in conjunction with high-frequency accelerations and decelerations, the multidomain stress profile amplifies the interpretive value of sweat cortisol. Atypically elevated cortisol during routine training may signal allostatic overload, while blunted responses during matches could flag HPA dysregulation—a potential harbinger of overtraining syndrome or adrenal insufficiency.

From a practical standpoint, sweat cortisol is presently the most advanced sweat-based hormonal marker for fatigue profiling in soccer, supported by preliminary data from soccer cohorts [31,49]. Future studies should prioritize concurrent sampling of sweat, plasma, and saliva for multi-matrix comparison, coupled with rigorous control of environmental and positional factors. Without such triangulation, the application of sweat biomarkers for fatigue diagnostics in soccer remains promising but premature.

### 3.4. Cytokines in Sweat and Inflammatory Fatigue

Interleukin-6 (IL-6), a pleiotropic cytokine with dual roles as a myokine and immunomodulator, is a central mediator in the physiological nexus linking metabolic stress, inflammation, and fatigue. During prolonged or high-intensity exercise, IL-6 is secreted by skeletal muscle fibers and immune cells, with plasma levels rising exponentially in response to glycogen depletion, tissue damage, and systemic immune activation. Centrally, IL-6 promotes sickness behavior—fatigue, lethargy, and reduced motivation—while peripherally mobilizing lipolytic and glucogenic reserves, making it a key mediator of both central and peripheral fatigue.

In soccer players, Souglis et al. (2018) demonstrated significant post-match surges in circulating IL-6, reflecting the sport’s repeated eccentric loading, neuromuscular strain, and metabolic cost [51]. However, while IL-6 is routinely quantified in blood across multiple sports, its utility as a sweat-derived biomarker in soccer remains underexplored. No current evidence exists for IL-6 quantification in sweat samples collected during or after elite soccer training or match play, underscoring a major translational gap.

In endurance athletes, IL-6 has been detected in sweat during prolonged exertion using absorbent patch-based assays, although its temporal kinetics diverge from plasma trends—sweat concentrations tend to decrease as exercise progresses, potentially due to glandular flushing or cortisol-mediated suppression of local secretion [51]. This decoupling complicates the interpretation of sweat IL-6 as a surrogate for systemic inflammation.

Mechanistically, the presence of IL-6 in sweat may originate from two potential sources: diffusion from systemic circulation and de novo synthesis by eccrine glands. Immunohistochemical studies confirm local glandular production of multiple cytokines—including IL-6, IL-1β, and TNF-α—raising questions about whether sweat IL-6 reflects systemic inflammation, localized immune activity, or a composite of both [39,40]. The molecule’s relatively large size (~20 kDa) limits passive diffusion from plasma, further muddying this distinction.

From a sport-specific perspective, sweat IL-6 remains unvalidated in soccer contexts. No current studies have mapped sweat IL-6 to post-match soreness, creatine kinase elevation, or perceptual fatigue indices in this population. Although intermittent sports such as basketball and tennis may provide indirect analogs, the unique physiological profile of soccer, characterized by a complex interplay of aerobic, anaerobic, neuromuscular, and cognitive stressors—necessitates tailored validation protocols.

Given IL-6’s dual mechanistic relevance and its inducibility by both metabolic and immunological load, it remains a theoretically compelling—but empirically unproven—candidate for non-invasive fatigue monitoring in soccer. Future research should employ simultaneous blood and sweat sampling under match-replicative conditions to establish kinetic concordance and functional thresholds predictive of recovery status and performance readiness.

Table 2 provides a critical appraisal of the current evidence supporting these candidate sweat biomarkers for fatigue monitoring in athletes. Although sweat biomarkers offer an alluring paradigm for non-invasive fatigue monitoring, current evidence is undercut by foundational methodological deficits that constrain their interpretive reliability. Chief among these is the paucity of gold-standard validation against concurrently sampled blood analytes. With few exceptions, studies fail to establish kinetic congruence between sweat and plasma dynamics during exercise, rendering inferences about internal physiological states speculative. For metabolites such as lactate and cortisol, sweat levels may parallel systemic trends under some conditions; yet, these relationships break down under variable intensities, thermoregulatory shifts, or metabolic flux.

A critical methodological limitation in sweat biomarker research lies in the anatomical heterogeneity of sweat gland distribution and function. Biomarker concentrations are highly dependent on sampling site due to regional differences in eccrine gland density, sympathetic innervation, and cutaneous microvascular perfusion. This is exacerbated by environmental confounders—ambient heat, humidity, and clothing insulation—which can independently distort sweat composition. Few studies standardize or report these variables, leaving biomarker trends susceptible to noise and poor reproducibility across protocols.

Moreover, sweat rate exerts a non-linear, often inversely proportional effect on solute concentration. High sweat rates dilute biomarkers such as lactate or ammonia, leading to misleadingly low values even as systemic levels climb. Without integrating sweat flow metrics or employing rate-normalized models, raw concentration data remains analytically compromised. For cytokines like IL-6, additional complexity emerges from their molecular weight (~20–25 kDa) and the dual origin hypothesis—transdermal migration versus local glandular synthesis. Immunohistochemistry has confirmed eccrine expression of IL-6 and TNF-α [39,40], suggesting that post-exercise elevations in sweat may not necessarily reflect systemic inflammation but instead local immuno-epidermal activation.

To date, no studies have validated sweat cytokine concentrations—such as IL-6—against established post-match inflammatory markers in soccer players, including serum creatine kinase (CK), C-reactive protein (CRP), or perceptual fatigue indices (soreness ratings). As a result, their translational relevance in this sport remains speculative. The existing literature is primarily extrapolated from endurance-based disciplines that differ substantially from soccer in terms of metabolic demands, mechanical loading patterns, and recovery kinetics. This mismatch between biomarker physiology and sport-specific context necessitates caution when generalizing findings across disciplines.

Robust application in elite soccer will require ecologically valid protocols that integrate simultaneous multi-matrix sampling (sweat, plasma, saliva), control for anatomical and environmental variance, and deploy wearable technologies capable of measuring both analyte concentration and sweat flux. Until such rigor is operationalized, sweat biomarkers for fatigue remain a promising but unvalidated domain.

## 4. Omics-Based Sweat Biomarker Profiling for Fatigue Monitoring

### 4.1. Sweat Metabolomics

Sweat metabolomics has identified many fatigue-related biomarkers, such as hypoxanthine (a purine metabolite from ATP breakdown), which rises in sweat after high-intensity exercise and can serve as a fatigue indicator. In a study involving intermittent athletes, upregulated sweat metabolites after fatigue included hypoxanthine, pyruvate, and amino acids, with downregulation of vitamin B6 derivatives and theophylline [30]. These metabolic shifts reflect alterations in purine turnover, glycolytic flux, and micronutrient utilization. Similarly, an untargeted sweat metabolomics study in soccer players found that nicotinate/nicotinamide (niacin) metabolism was altered by exercise [31]. Nicotinic acid (vitamin B3), which is a precursor for NAD/NADP, was the only metabolite significantly associated with fatigue. Niacin was highly abundant before exercise and dropped post exercise, highlighting its role in redox energy supply.

Pathway enrichment analysis of sweat profiles implicates amino acid and energy metabolism in fatigue. In soccer players, key altered pathways included alanine/aspartate/glutamate metabolism, branched-chain amino acid degradation, and nicotinate/nicotinamide metabolism. These reflect increased protein breakdown and NAD cycling during intense play. Other metabolites frequently observed in sweat include lactate, ammonia, urea, creatinine, and various organic acids (pyroglutamate). Notably, sweat ammonia and urea indicate protein catabolism, while branched-chain amino acids (BCAAs) (valine, isoleucine) drop as they fuel exercise. Such sweat metabolic patterns mirror muscle and systemic fatigue status.

Recent innovations also tap sweat extracellular vesicles (EVs). EVs are lipid-bound particles carrying metabolites. One study isolated EVs from sweat patches during exercise and recovery, identifying 17 EV-associated metabolites (amino acids, glutamate, glutathione, fatty acids, creatine, glycolysis intermediates) [59]. These EV-metabolites showed distinct profiles in exercise vs. recovery, suggesting EV-metabolomics as a “next-generation” sweat biomarker source.

### 4.2. Sweat Proteomics and Inflammatory Markers

Historically and practically, proteomic analysis of sweat has lagged behind other biofluids due to the inherently low protein concentration in eccrine secretions. The limited protein content is often dominated by skin-derived contaminants and innate defense molecules. Nevertheless, targeted assays in sweat have detected signaling molecules (cytokines, hormones) relevant to fatigue [60]. Cytokines such as interleukin-6 (IL-6) and tumor necrosis factor-α (TNF-α) can be measured in sweat and rise after prolonged exercise. These reflect systemic inflammation and may correlate with overtraining or recovery status. Some studies have even quantified neuropeptides or hormones (neuropeptide Y, cortisol) in sweat. A cortisol-imprinted patch achieved high-sensitivity cortisol detection in sweat for stress monitoring. Such hormone sensors illustrate that sweat proteomic/peptidomic biomarkers (stress or immune mediators) are now accessible via high-sensitivity wearable assays and mass spectrometry.

### 4.3. Sweat Metallomics/Mineralomics

As discussed previously, electrolyte levels in sweat are exercise- and individual-dependent. In one study of professional soccer players, forearm sweat contained ~26.7 ± 11.3 mM Na^+^ and athletes lost ~43 ± 15.9 mmol Na^+^ in 90 min training [32]. Sustained high sweat rates (>1.5 L/h) lead to substantial Na^+^ and K^+^ losses. Since >2% dehydration impairs performance, monitoring sweat Na^+^/Cl^−^ helps manage hydration. Studies report that sweat Na^+^ and Cl^−^ are physiologically lower than plasma due to reabsorption in sweat ducts, whereas K^+^, ammonia, urea, and lactate are typically higher in sweat than in blood [33]. In sequential exercise bouts, Na^+^, K^+^, Ca^2+^, and Mg^2+^ excretion stayed fairly constant, but Zn loss dropped.

Physiologically, trace metals and minerals in sweat can reflect systemic status. Prolonged exercise markedly alters mineral metabolism resulting in significant losses of Mg, Zn, Cu, Fe, and Cr. Typical sweat contains ~3–4 mg/L Mg^2+^ (plasma Mg often falls after hard work), and Zn appears at ~0.5–1.0 mg/L sweat [34]. Endurance exercise can cause multi-milligram losses of these elements (~12 mg Mg per hour in heavy sweating), potentially leading to deficiency over time. Tang et al. (2016) found that strenuous exercise drove excretion of heavy metals in sweat; for example, chromium, copper, zinc, cadmium, and lead were all measurable in sweat, with significantly higher levels in sweat than in urine after exercise [61]. While heavy metals (Cd, Pb) are more of a detoxification interest than fatigue biomarkers, their sweat levels underscore the breadth of analytes accessible via sweat.

**Figure 2:**
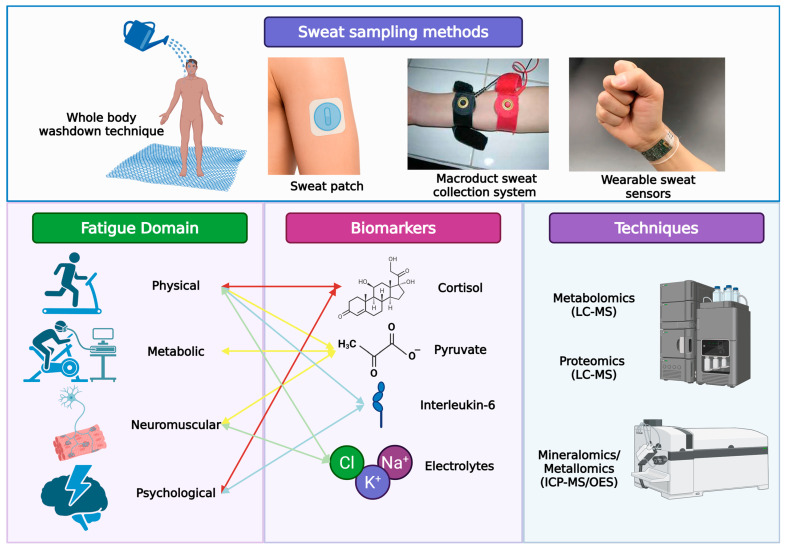
Sweat sampling methods, fatigue domains, biomarkers, and analytical techniques relevant to athlete fatigue monitoring. The upper panel illustrates primary sweat sampling approaches including whole-body washdown, sweat patches, Macroduct collectors, and wearable sweat sensors. The lower panel conceptually maps key fatigue domains (physical, metabolic, neuromuscular, psychological) to candidate sweat biomarkers (cortisol, pyruvate, interleukin-6, electrolytes).

These analytes and their corresponding analytical methodologies are summarized in Table 3. While omics-based profiling provides powerful analytical tools to identify and quantify sweat biomarkers, their practical utility in soccer depends critically on effective, field-ready sampling methods capable of capturing representative sweat data under real-world conditions (Figure 2).

## 5. Sweat Sampling Methods in Soccer

In soccer, sweat monitoring is critical for individualizing hydration strategies given high-intensity intermittent exercise and variable environments [39,40]. The whole-body washdown (WBW) method remains the laboratory gold standard for measuring total sweat volume and electrolyte composition. This technique involves rinsing the entire body with deionized water post exercise to capture all sweat. Though highly accurate, it is impractical for field settings due to its complexity and equipment requirements.

For field-based practice, soccer research overwhelmingly relies on absorbent patch sampling. Patches (gauze or filter pads) are applied to multiple skin sites (chest, back, arm, thigh) to capture localized sweat during training or matches. After removal, sweat is extracted for electrolyte analysis. This approach is simple, minimally invasive, and has been widely used in studies of both male and female soccer players to measure sweat sodium and potassium concentrations. However, sweat composition varies by body region, requiring multiple sites or pooling to approximate whole-body values [11,12,13,14,15]. Sweat rate is typically estimated via gravimetric body mass change before and after exercise, corrected for fluid intake and urine output. This method is highly practical and standard in soccer settings to quantify sweat volume losses, which typically range from 0.5–2 L/h, sometimes exceeding 2.5 L/h in hot climates.

Recent innovations include wearable microfluidic patches and electronic sweat sensors. Microfluidic platforms direct sweat into micro-reservoirs, where colorimetric or electrochemical sensors quantify analytes—such as chloride—continuously during exercise. Validation studies have demonstrated high concordance with traditional patch methods, while enabling dynamic assessment of hydration status. Electronic sensors further enable on-demand sweat stimulation via iontophoresis and real-time tracking of electrolytes and metabolites such as lactate and cortisol [11,12,13,14,15]. These cutting-edge wearables offer soccer practitioners real-time, personalized hydration guidance and represent a major step toward integrated, automated monitoring in elite sport.

### Wearable Sweat Sensor Technologies

To operationalize real-time monitoring of sweat biomarkers, a new generation of flexible wearable sensors is being developed and commercialized. These systems integrate microfluidic architecture with electrochemical sensing technologies to provide continuous, non-invasive assessment of sweat composition [12,35]. Common platforms use enzyme electrodes (lactate oxidase, glucose oxidase) and ion-selective electrodes (ISEs) built into skin patches, bands, or textiles. An integrated patch was reported with enzymatic electrodes for lactate and glucose and ISEs for Na^+^ and K^+^, along with a temperature sensor. Such multiplexed systems enable simultaneous monitoring of energy metabolites and electrolytes. Electrochemical sweat sensors have been demonstrated for lactate (using LOx), glucose, alcohol, urea, ${\mathrm{NH}}_{4}^{+}$, pH, and metal ions (Na^+^, K^+^, Ca^2+^). Among these, amperometric lactate sensors are particularly well-validated, displaying sweat lactate kinetics that parallel blood lactate responses and demonstrating sensitivity to exercise intensity. Recent devices even use biofuel cells such as a screen-printed patch combining a lactate biofuel cell with a colorimetric Cl^−^ sensor, capturing sweat volume, pH, lactate, glucose, and chloride simultaneously. Other examples include textile-based gold-fiber lactate sensors, and NFC/battery-free patches for multiplex sweat panels.

Sweat collection methods vary, with techniques such as Macroduct^®^ collectors and adhesive patches commonly employed. In a recent soccer study, players wore eight adhesive-free Macroduct© devices on arms, collecting ~80 µL sweat each during a 20 min high-intensity run [31,62]. These portable devices enabled concurrent measurement of accelerometry data, RPE scales, and sweat metabolites. Emerging microfluidic patches route sweat to sensor chambers without manual transfer. Advanced platforms utilize flexible printed circuits, hydrogels, and molecularly imprinted polymers for robust on-skin analysis [35,36].

The current technological readiness and sport-specific applicability of key sweat biomarkers for fatigue monitoring in elite soccer are summarized in Table 4, which outlines their physiological relevance, analytical limitations, and stages of validation—from controlled laboratory studies to real-world field deployment.

## 6. Key Gaps in Sweat Biomarker Research for Elite Soccer Players

Current research on sweat biomarkers in elite soccer populations remains constrained by significant methodological, contextual, and demographic limitations. Most extant studies are limited by small, homogeneous samples—typically involving 20 to 30 athletes from a single club, league, or climatic region—thereby undermining generalizability and translational relevance. Maughan et al. (2004) investigated 24 Premier League players in controlled training environments [63], while Suarez-Ortegón et al. (2024) evaluated 32 Colombian professionals outside match-play contexts [32]. Critically, the majority of studies are confined to laboratory-based ergometry or pre-season conditioning protocols, failing to replicate the intermittent, multidirectional loading patterns, psychophysiological arousal, and environmental variability inherent to competitive match-play. This absence of ecological validity represents a major limitation. Furthermore, longitudinal monitoring across an entire competitive season is virtually absent, precluding meaningful insights into cumulative fatigue trajectories, recovery patterns, or overtraining risk—particularly in elite youth populations, who are more susceptible to chronic under-recovery syndromes.

Significant methodological heterogeneity further impairs interpretability. Sweat patch placement varies inconsistently—from deltoid to upper back to thigh—with no consensus on anatomical standardization. Calibration procedures, including correction for sweat rate, local humidity, or glandular density, are inconsistently reported or omitted entirely, raising significant concerns regarding reproducibility and cross-study comparability. The absence of validated standard operating procedures for sweat collection, stimulation, and stabilization compromises both intra- and inter-study reliability.

Biomarker-specific validation in soccer-specific contexts remains strikingly underdeveloped. Although wearable platforms can reliably detect sweat lactate, its correspondence with blood lactate under soccer conditions is equivocal. Several studies demonstrate inverse kinetics at higher workloads—possibly due to dermal glycolysis, sweat rate dilution, or local pH effects—challenging the assumption of linear extrapolation [64]. Similarly, sweat ammonia exhibits high inter- and intra-individual variability. Although blood ammonia consistently rises with increasing metabolic strain, sweat ammonia concentrations have been observed to decline at higher intensities, likely due to sweat rate-induced washout or pH-dependent solubility effects [65]. Cortisol, a canonical biomarker of psychophysiological stress, lacks validated normative thresholds or known diurnal patterns in sweat during match-play, rendering real-time interpretation speculative at best. Inflammatory cytokines such as IL-6 remain largely undetectable post-exercise in sweat across several studies [66], calling into question their suitability as fatigue indices under current assay limitations. Sweat electrolyte measurements, while more established in clinical diagnostic paradigms (cystic fibrosis), poorly correlate with plasma levels during exercise due to site-dependent glandular secretion variability and evaporative losses [42]. Emerging targets such as hypoxanthine and pyruvate—both implicated in systemic metabolic stress and oxidative perturbation—have been identified in sweat metabolomics of endurance athletes but remain wholly unexamined in soccer, leaving their diagnostic sensitivity and contextual validity unknown [30].

Temporal and ecological limitations further constrain current understanding. Nearly all studies to date are cross-sectional, acute investigations, lacking repeated-measures designs needed to elucidate intra-individual fatigue kinetics over time. In-match sweat sampling—despite its high ecological validity for team sports—is almost entirely absent. Most protocols rely exclusively on pre- and post-session values, thereby missing critical within-session fluctuations during halves, substitutions, tactical stoppages, or periods of acute thermoregulatory strain. Very few studies employ multiplexed or multi-domain analysis (co-tracking metabolic and endocrine markers), precluding systems-level interpretation of fatigue states.

Demographically, the literature remains heavily male-centric and confined to temperate-climate, high-resource settings. Female athletes are profoundly underrepresented [67], and youth athletes are seldom studied beyond hydration screening [68]. This omission disregards critical sex-based physiological differences—including hormonal fluctuations across the menstrual cycle that influence sweat rate, thermoregulation, and substrate utilization. Estrogen and progesterone modulate carbohydrate-to-lipid fuel shifts, which may manifest in altered sweat metabolite profiles. Furthermore, female players exhibit distinct neuromuscular fatigue kinetics and elevated susceptibility to soft-tissue injuries such as ACL rupture—factors potentially mediated by cytokine and glucocorticoid dynamics. Without sex- and age-specific biomarker validation, sweat-omics in soccer risks both reduced sensitivity and potential misinterpretation, especially in developmental cohorts.

Despite growing scientific interest, the translation of biomarker research into field-deployable, sweat-based monitoring platforms for elite soccer remains nascent. Systematic reviews, including that by Soler-López et al. (2024), highlight a predominance of biomarker research rooted in laboratory-based blood or saliva assessments, with scant exploration of wearable, non-invasive modalities in real-world team sport settings [69]. Devices optimized for static or cyclical modalities (cycling, treadmill running) often fail under high-movement, high-contact, sweat-saturated, and climatically variable match conditions. Common limitations include compromised adhesive integrity, signal artifacts from collisions, and sweat pooling due to occlusive textiles. Critically, most current sensors lack dynamic compensation for sweat rate, skin temperature, and regional glandular variation—resulting in uncorrected signal noise and reduced validity in field settings.

Equally problematic is the disconnect between raw biomarker data and its operational interpretation by sport science practitioners. Unlike external load metrics (GPS, heart rate) with well-characterized interpretive thresholds, sweat biomarkers remain analytically ambiguous in soccer. Interpreting fluctuations in sweat lactate, ammonia, cortisol, or IL-6 in real time requires complex multivariate normalization algorithms capable of adjusting for inter-individual baselines, intra-session kinetics, sweat rate dynamics, and circadian fluctuations. No current commercial or research platforms offer integrated decision-support systems that can combine biomarker output with contextual variables such as tactical load, substitution patterns, perceived exertion, or environmental stress. Development of real-time dashboards with embedded machine-learning algorithms trained on soccer-specific data is therefore essential to bridge this translational gap.

Human-factor considerations also present critical barriers to widespread adoption. Athlete adherence may be limited by discomfort, skin irritation, movement restriction, or concerns about over-surveillance. Device bulk, aesthetic obtrusiveness, adhesive reliability under friction, and sensory burden during prolonged activity all affect user acceptability. Furthermore, the highly individualized nature of sweat biomarker data raises legitimate concerns regarding data privacy, ethical use, and biometric ownership. Without clearly defined governance frameworks addressing these legal, psychological, and ethical dimensions, uptake within elite sport settings will remain limited.

Despite these multifactorial barriers, technological and methodological pathways toward resolution are emerging. Integration of microfluidic sweat rate sensors—such as thermal mass flow detectors or electrochemical conductance modules—into on-body platforms may allow for real-time concentration normalization. Concurrently, an emerging anatomical consensus supports the forearm and upper back as optimal sweat collection sites, offering a balance between eccrine gland density, sensor retention, and movement stability. Calibration strategies involving dual matrix (sweat–blood) regression mapping are under pilot, with the goal of establishing valid conversion functions for field application. Protocols standardizing sweat stimulation, storage, and analyte stabilization are in development to reduce inter-protocol variability and improve cross-study comparability. Advances in flexible biosensor materials and multi-analyte microfluidic platforms further support the feasibility of continuous, multidomain fatigue profiling during actual match-play.

Importantly, current research overwhelmingly emphasizes acute, session-level fatigue monitoring yet neglects the longitudinal dimension critical for injury prevention and athlete development. Overtraining syndrome (OTS)—a maladaptive state of chronic under-recovery associated with sustained performance decline, immune suppression, and mood disturbance—remains a central concern in elite youth soccer, where biological immaturity intersects with high competitive loads. To date, the longitudinal applicability of sweat-omics for detecting chronic elevations in cortisol, interleukin-6 (IL-6), or exercise-induced metabolic by-products remains entirely unexplored in soccer-specific contexts. Future research must prioritize season-long, repeated-measures biomarker tracking to identify maladaptive recovery trajectories, establish normative variation bounds, and validate sweat-omics as a non-invasive surveillance tool for early OTS detection and prevention.

## 7. Recommendations for Integrative Fatigue Monitoring Framework for Soccer Practitioners

To translate sweat-based biomarker research into applied sports science settings, an integrated multidimensional framework is required, which triangulates biochemical indicators with established external and internal load monitoring systems. In practice, sweat biomarkers should complement, not replace, traditional fatigue surveillance tools such as GPS-derived locomotor metrics (total distance, high-speed running, accelerations), heart rate (HR)-based internal load indices (training impulse [TRIMPs]), and subjective psychometric tools (Ratings of Perceived Exertion [RPEs], Total Quality Recovery [TQR], and the Hooper Index).

An integrated monitoring model may encompass the following components:

- Pre- and post-session sweat sampling via wearable microfluidic sensors for markers like lactate, cortisol, and Na^+^/Cl^−^ losses.
- Real-time GPS and HR tracking to quantify acute mechanical and cardiovascular load.
- Daily subjective assessments for mood, sleep, muscle soreness, and perceived exertion.
- Cross-sectional validation by correlating biochemical fluctuations with known fatigue events (spike in high-intensity distance, congested match fixtures, menstrual phase in females).
- Contextual interpretation wherein elevated sweat cortisol or IL-6, in tandem with declining subjective wellness and unaltered external load, might signal cumulative stress not visible in GPS data alone.

While GPS and HR provide surface-level outputs of external and cardiovascular work, sweat biomarker data offers a unique window into the underlying biochemical strain-reflecting immune, endocrine, and metabolic status. In elite environments, these inputs should be integrated into individualized recovery decision-making matrices, informing rotation, training modifications, or recovery interventions. This approach is particularly pertinent in youth academies and women’s football programs, where the use of non-invasive, ecologically valid methods (sweat-based monitoring over blood sampling) may enhance feasibility and athlete compliance. Future protocols should aim to automate such integration within practitioner dashboards, enabling longitudinal load-fatigue-recovery modeling rooted in real-time data streams.

## 8. Future Perspectives

Future research should focus on validating sweat-based biomarkers in real match conditions, particularly across different stages of fatigue accumulation and recovery. A critical objective is the identification of biomarker thresholds corresponding with key performance inflection points, such as the psychomotor fatigue threshold—the point at which cognitive–motor integration degrades due to cumulative physiological and psychological load [70,71]. Identifying sweat analytes that reliably track this threshold could enable early detection of declines in decision-making, reaction time, and neuromuscular coordination.

Moreover, personalized sweat profiling is essential, accounting for inter-individual variability in sweat rate, composition, and responsiveness to training stressors. However, a persistent and concerning gap in the literature is the near-total exclusion of female and youth athlete cohorts, particularly in soccer. This omission not only limits the ecological validity of current biomarker models but risks perpetuating sex- and age-specific misinterpretations of fatigue physiology.

Future studies must adopt longitudinal, developmentally informed designs within elite youth academies, especially those supporting female players, to chart ontogenetic trajectories of sweat biomarker expression across pubertal stages and varying training loads. At present, no longitudinal sweat biomarker studies currently exist in soccer that track fatigue accumulation across training blocks, congested match calendars, or recovery cycles.

The potential application of sweat-omics for OTS surveillance—a syndrome with significant consequences for youth player development—remains entirely speculative without field-validated temporal profiling frameworks. Thus, sweat-omics must evolve from isolated session-level snapshots to temporally integrated fatigue diagnostics. Longitudinal validation across training microcycles, particularly for OTS-related endocrine and inflammatory pathophysiology, should be prioritized. Given the known modulatory effects of endogenous hormonal rhythms, particularly the menstrual cycle, on thermoregulation, sweat gland output, and immune signaling, controlled investigations into intra-cycle variability in sweat analytes (cortisol, IL-6, electrolytes) are urgently warranted. Furthermore, field-based validation using wearable biosensors must be implemented during live training and match play, correlating sweat biomarker data with perceptual exertion, neuromuscular recovery status, and objective performance decrements. Integration with GPS, HR, and movement analytics would further enhance predictive model fidelity. Only through such rigorous, population-specific inquiry can sweat biomarkers be credibly integrated into female and youth athlete monitoring frameworks.

Ultimately, the goal is to realize a multi-modal, real-time fatigue monitoring system that is accessible, non-invasive, and actionable in elite sport environments.

## 9. Conclusions

Sweat-based biomarker monitoring is an exciting frontier in sports science for assessing fatigue across multiple domains. The physiological and biochemical information contained in sweat, from metabolites like lactate and ammonia to electrolytes, hormones, and cytokines, provides a composite picture of an athlete’s exertion and stress levels. This is particularly valuable in elite sports such as soccer, where performance and recovery are finely balanced. By correlating sweat biomarkers with fatigue domains, practitioners can non-invasively gauge metabolic fatigue (high lactate indicating anaerobic stress), neuromuscular fatigue (salt losses risking cramps), psychological fatigue (elevated cortisol reflecting stress), and thermal fatigue (high sweat rate and dehydration). Early adoption in soccer and endurance sports has demonstrated the practicality of this approach, especially with wearable sweat-sensing technology that allows for real-time data capture. That said, challenges remain in interpreting sweat data because sweat composition is influenced by many factors (glandular mechanisms, skin contamination, sensor calibration), and further research is needed to refine the links between sweat metrics and internal fatigue states. Nonetheless, the current literature is encouraging and show that sweat biomarkers have clear relationships with known fatigue processes, as initial field studies validate their relevance in athletic settings. In the coming years, we can expect sweat diagnostics to become a staple in athlete monitoring systems, enabling coaches and sports medicine professionals to detect fatigue early, personalize recovery protocols, and ultimately safeguard athlete health while maximizing performance.

## Figures and Tables

**Table 1:** Conceptual mapping of fatigue domains in elite soccer to candidate sweat biomarkers and detection methods.

Fatigue Domain	Mechanism	Candidate Sweat Markers	Detection Method(s)
Peripheral/muscular	Muscle metabolite accumulation (ATP depletion, H^+^ accumulation) [29]	Lactate, ammonia, ${\mathrm{NH}}_{4}^{+}$, H^+^, BCAAs (alanine, valine) [30,31]	Metabolomics (GC-MS/LC-MS) [30,31], wearable lactate/ammonia sensors
Central Nervous System (CNS)	Reduced motor drive, neurotransmitter changes	Cortisol, neuroactive metabolites	Sweat cortisol immunosensors, untargeted metabolomics
Cognitive/mental	Sustained attention, stress	Cortisol, cytokines (IL-6, TNF-α)	As above, plus potential multiplex cytokine assays
Thermal/ Dehydration	Heat stress, fluid/electrolyte loss [32]	Na^+^, Cl^−^, K^+^, Mg^2+^, Zn (electrolyte imbalances) [33,34]	Ion-selective electrodes (sweat Na^+^, K^+^ sensors) [35], ICP-MS or ion chromatography
Overtraining/Chronic	Immune/endocrine dysregulation	Cytokines, cortisol, creatine kinase (indirect)	Sweat immunoassays (cortisol ELISA) [36], proteomics

**Table 2:** Critical appraisal of candidate sweat biomarkers for athlete fatigue monitoring.

Biomarker	Evidence Quality	Sample Sizes	Populations	Validity	Conflicts	References
Lactate	Moderate	Typically *n* = 5–15	Healthy adult exercisers; no youth soccer data	Wearable sensors show rising sweat lactate with exercise intensity; partially mirrors exertion but not 1:1 with blood lactate	Variable correlation to blood lactate; local skin glycolysis contribution	[16,52,53]
Ammonia	Weak	~10–20 in pilot studies	Adults in lab exercise protocols	Electrochemical patches show sweat ammonia sensing feasible; high variability; sweat rate dependence	Conflicting lab results: some show increased, others decreased sweat ammonia with blood rises	[39,40,54]
Cortisol	Moderate	10–30 participants	Healthy young adults; no youth data	Wearable patches demonstrated good correlation to blood/saliva cortisol in pilots; sensitive detection achieved	Influenced by sampling site, sweat rate; small samples	[28,55,56]
IL-6	Weak	20–50 in lab studies	Adults, older vs. younger comparisons; no sport field studies	Detectable in sweat with ultra-sensitive assays; no wearable on-body sensors yet	Sweat IL-6 may reflect local skin, not systemic inflammation	[57,58]
Electrolytes (Na^+^, K^+^, Cl^−^)	Strong (for Chronic Fatigue (CF) diagnosis); Low (for hydration/fatigue monitoring)	Thousands in clinical CF data; 10–50 in sport hydration studies	CF patients; endurance athletes; limited soccer-specific data	Ion-selective wearables validated for sweat Na^+^/K^+^ in sport settings; real-time feasible	Hydration biomarkers vary with sweat rate, acclimation; limited correlation to blood	[13,53]
Hypoxanthine	Very Low	n = 14 in metabolomics pilot	Adult runners	LC-MS detects sweat hypoxanthine post-exercise; lab-only	No wearable sensors; no real-time field data	[30]
Pyruvate	Very Low	n = 14 in same pilot as above	Adult runners	Lab assays detect rise post-high intensity exercise	No wearable sensors; no blood correlation studies	[30]

**Table 3:** Summary of sweat-omics analytical approaches for fatigue biomarker profiling.

Omics Approach	Targets	Techniques/Equipment	References
Metabolomics	Small molecules (lactate, amino acids, hypoxanthine)	GC-MS, LC-MS (GC/GC-TOF-MS), NMR, targeted biosensors	[30,31]
Proteomics/Peptidomics	Proteins/cytokines (IL-6, cortisol, defensins)	LC-MS/MS proteomics, immunoassays, electrochemical biosensors (MIP cortisol sensor)	[36,60]
Metallomics/Ionomics	Electrolytes (Na^+^, K^+^, Cl^−^, Mg^2+^, Ca^2+^), trace metals (Zn, Cu, Fe)	Ion chromatography, ICP-MS, wearable ISE sensors	[34,35]
Extracellular Vesicle Metabolomics	EV cargo metabolites (fatty acids, amino acids)	EV isolation (ultracentrifugation/pads) + targeted MS	[59]

**Table 4:** Technology Readiness Levels of key sweat biomarkers for fatigue monitoring in elite soccer.

Biomarker	Readiness Tier	Description	References
Lactate	Pilot-stage (High TRL*, nearing field-ready)	Wearable sweat sensors demonstrated on athletes with good correlation to blood lactate. Used in controlled exercise tests and training trials. Limited robust validation in full matches.	[52,53]
Ammonia	Pilot-stage (Mid TRL)	Early electrochemical patches show feasibility during exercise. Tested in lab/short trials; not yet validated in field training or soccer matches.	[53,54]
Cortisol	Pilot-stage (Mid TRL)	Wearable patches demonstrated cortisol tracking during exercise. Good lab validity but limited field deployment. Needs better stability for match settings.	[53,55]
IL-6	Lab-stage (Low TRL)	Only lab-based detection using advanced assays. No wearable or on-body sensors. No field validation in sport contexts.	[53,57]
Electrolytes (Na^+^, K^+^, Cl^−^)	Field-ready (High TRL)	Multiple wearable sensors validated in sport training (running, cycling). Some commercial products available. Proven use in hydration planning.	[39,40,53]
Hypoxanthine	Lab-stage (Low TRL)	Identified via sweat metabolomics post-intense training. No wearable sensor exists; analysis limited to lab LC-MS.	[30]
Pyruvate	Lab-stage (Low TRL)	Detected rising in sweat post-high-intensity intervals in lab studies. No wearable sensors; measured only offline.	[30]

Note: *TRL stands for Technology Readiness Level. Readiness tiers were assigned based on multi-tiered criteria reflecting the development and deployment maturity of each biomarker monitoring system: lab-stage (TRL 2–4) indicates conceptual or benchtop validation without on-body or field testing; pilot-stage (TRL 5–6) includes initial prototype testing in controlled trials with limited sports-specific data; field-ready (TRL 7–9) denotes validated use in athletic contexts with demonstrated robustness in training or match conditions. Criteria were adapted from published TRL frameworks contextualized for wearable biosensor deployment in sport [53,54].

## Data Availability

No new data was created in this study.
